# Cornelia de Lange Syndrome: A Newborn with Imperforate Anus and a *NIPBL* Mutation

**DOI:** 10.1155/2012/247683

**Published:** 2012-12-10

**Authors:** Rose H. Mende, David P. Drake, Raimos M. Olomi, Ben C. J. Hamel

**Affiliations:** ^1^Department of Paediatrics and Child Health, Kilimanjaro Christian Medical Centre, P.O. Box 2240, Moshi, Tanzania; ^2^Department of Paediatrics and Child Health, Kilimanjaro Christian Medical University College, P.O. Box 2240, Moshi, Tanzania; ^3^Department of Paediatric Surgery, Hospital for Sick Children, Great Ormond Street, London WC1N 3JH, UK; ^4^Directorate of Postgraduate Studies, Kilimanjaro Christian Medical University College, P.O. Box 2240, Moshi, Tanzania

## Abstract

Cornelia de Lange syndrome is a dominantly inherited, genetically heterogeneous and clinically variable syndrome with multiple congenital anomalies and developmental delay. Gastrointestinal anomalies are common and an important cause of morbidity and mortality. We report on a newborn with a molecularly confirmed Cornelia de Lange syndrome who had an imperforate anus. This is the third report of Cornelia de Lange syndrome and imperforate anus.

## 1. Introduction

Cornelia de Lange syndrome (CdLs) (OMIM no. 122470, 300590, and 610759) is a well-known, dominantly inherited, variable syndrome with multiple congenital anomalies and developmental delay. It is a mainly characterized by craniofacial, limb and growth anomalies, and motor and intellectual disability. A variety of other anomalies (gastrointestinal, cardiac, renal, genital, and ocular) are often also present. Prevalence is estimated to be as high as 1/10,000 [1]. In a classical case, clinically, the diagnosis is not difficult, but in mild cases it might be more challenging. CdLs is genetically heterogeneous. In more than 50% of cases, a heterozygous, mostly de novo, mutation can be detected in one of 3 cohesin structural components encoding genes *NIPBL *(5p; ~50%), *SMC1A* (10q; ~5%) and *SMC3* (Xp; <1%; hemizygous), while recently mutations in a 4th related gene, *HDAC8* (Xq), were identified (OMIM no. 300882) [2]. In a further related gene, *RAD21* (8q) mutations were found in a mild CdLs phenotype (OMIM no. 614701) [3].

A number of CdLs reviews, highlighting clinical features and genotype-phenotype correlations, are available in the literature [4–9].

We report a case with an imperforate anus, in whom a *NIPBL* mutation was detected.

## 2. Case Report

**Figure 1:**
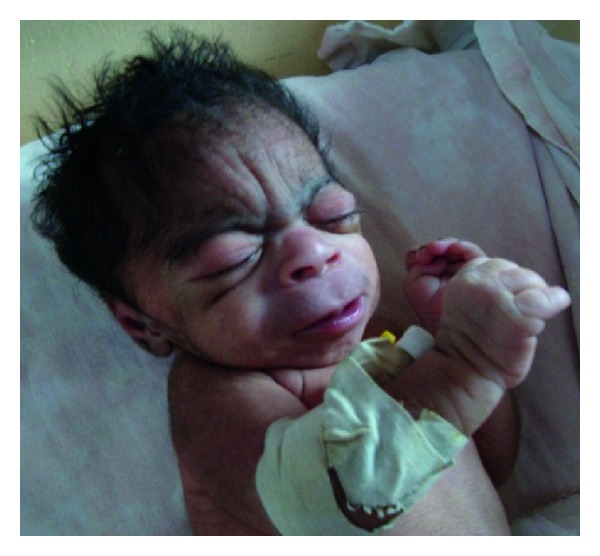
The characteristic craniofacial features of Cornelia de Lange syndrome.

**Figure 2:**
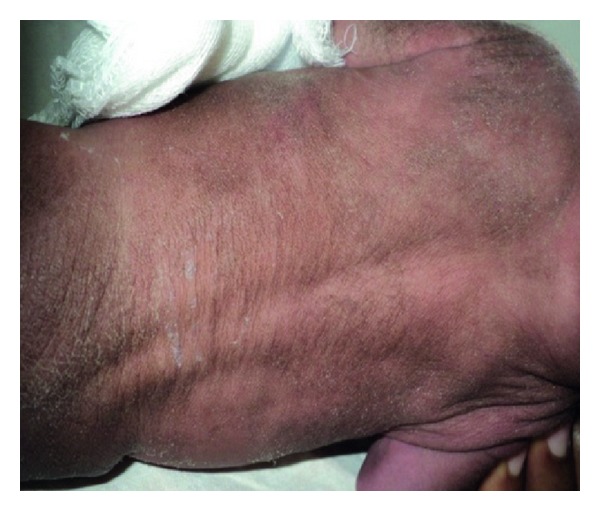
Generalized hirsutism.

**Figure 3:**
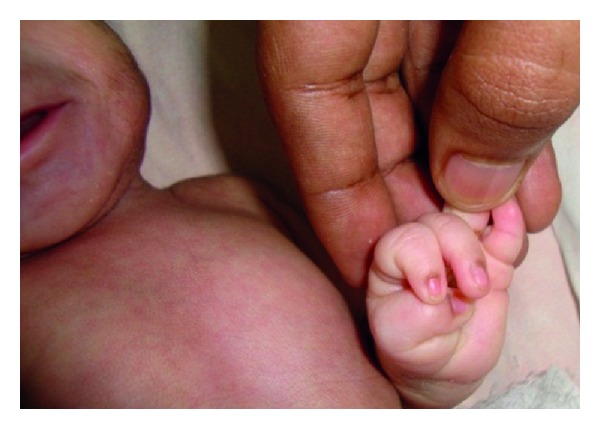
Clinodactyly of left fifth finger.

A one-day-old male newborn was referred to our hospital due to an imperforate anus. He was born vaginally after an uneventful pregnancy, apparently full term, with a birth weight of 1800 gr and an OFC of 29 cm, both far below the 2nd percentile. His nonconsanguineous parents are healthy as are his 5 siblings. Dysmorphic features were immediately noted: hirsute forehead, synophrys, bushy and arched eyebrows, long eyelashes, depressed nasal bridge, thin upper lip with down-turned corners, long philtrum, and micrognathia (Figure 1). Furthermore, generalized hirsutism was seen (Figure 2), as well as clinodactyly of the left fifth finger (Figure 3), limited elbow extension, and coronal hypospadias. The imperforate anus was confirmed. He had a weak sucking reflux and a weak cry.

Chest X-ray and echocardiography were normal. Abdominal ultrasound revealed moderate right-sided hydronephrosis, while the left kidney could not be visualized.

A colostomy was performed. He was discharged in a fair condition after 2 weeks and regular followup was initiated.

Venous blood was sampled, and DNA was sent to the Netherlands (DNA Diagnostic Laboratory, Department of Clinical Genetics, Amsterdam Medical Centre, Amsterdam). Sequence analysis of the *NIPBL* gene revealed a heterozygous nonsense mutation (c.3445C>T), leading to a premature stop codon in the protein (p.Arg1149X), thereby confirming the clinical diagnosis of CdLs.

## 3. Discussion

Due to the presence of the classical craniofacial features and prenatal growth retardation, there was a little doubt that CdLs was the correct diagnosis, though the limb anomalies were mild. The nonsense mutation in the *NIPBL* gene leading to a truncated protein confirmed the diagnosis. Though DNA of the healthy parents was not analysed, it is reasonable to assume that the mutation occurred de novo, though germline mosaicism cannot be excluded. The c.3445C>T mutation has not been described before. However, the great majority of *NIPBL* mutations found so far are private mutations, while a small minority are recurrent mutations [5, 6, 8].

Gastrointestinal anomalies are frequent in CdLs and an important cause of morbidity and mortality [9, 10]. The commonest is gastrooesophageal reflux with its sequelae [11]. Less frequent among others are pyloric stenosis, malrotation with volvulus, congenital diaphragmatic hernia, and small bowel duplication. Though hundreds of cases of CdLs have been described, to our knowledge, imperforate anus has only been reported twice: one in a Korean case and one in an overview of causes of mortality in CdLs [9, 12].

In conclusion, imperforate anus is an infrequent feature of CdLs, which should be added to the list of CdLs features in databases and textbooks.
